# DAYS SPENT AT HOME BEFORE AND AFTER HIP FRACTURE AMONG MEDICARE BENEFICIARIES LIVING WITH DEMENTIA: A COHORT STUDY

**DOI:** 10.1093/geroni/igad104.1333

**Published:** 2023-12-21

**Authors:** Jason Falvey, Chixiang Chen, Abree Johnson, Kathleen Ryan, Michelle Shardell, Haoyu Ren, Lisa Reider, Jay Magaziner

**Affiliations:** University of Maryland School of Medicine, Baltimore, Maryland, United States; University of Maryland, Baltimore, Maryland, United States; University of Maryland, Baltimore, Maryland, United States; University of Maryland, Baltimore, Maryland, United States; University of Maryland School of Medicine, Baltimore, Maryland, United States; University of Maryland Baltimore County, Baltimore, Maryland, United States; Johns Hopkins Bloomberg School of Public Health, Baltimore, Maryland, United States; University of Maryland Baltimore, Baltimore, Maryland, United States

## Abstract

Hip fracture is a disabling event experienced disproportionately by older adults with Alzheimer’s disease or related dementias (ADRD). Understanding how to better prognosticate outcomes for this population could drive more tailored clinical decision making. Our objective was to identify distinct trajectories of health before a hip fracture and determine their influences on post-fracture recovery trajectories and 1-year mortality rates among older adults with Alzheimer’s disease or related dementias (ADRD). We conducted a cohort study of 16,576 community-dwelling Medicare beneficiaries living with ADRD who experienced hip fracture between 2010 and 2017. We used latent mixture modeling to evaluate trajectories of days at home assessed monthly up to 180 days prior to hospitalization, and their associations with post-fracture days at home and 1-year mortality. Before a hip fracture, 3 distinct trajectories were identified: Robust (n=14,980, 90.3%), Impaired but Improving (n=809, 5.3%), and Impaired and Declining (n=787, 4.7%). Membership in the Impaired and Declining pre-fracture trajectory was strongly associated with membership in less favorable post-fracture recovery trajectories and 65% higher 1-year mortality rate (hazard ratio 1.65, 95% confidence interval 1.45-1.87) as compared to those in the Robust trajectory group. Similar albeit weaker associations were observed between membership in the Impaired but Improving pre-fracture trajectory and post-fracture outcomes. Overall, our results indicate that pre-fracture days at home trajectories are highly associated with post-hospitalization outcomes among older adults with ADRD and could be used to guide care management decisions.

